# The incidence of nephrogenic systemic fibrosis in subjects receiving gadoversetamide for cardiovascular magnetic resonance

**DOI:** 10.1186/1532-429X-14-S1-P48

**Published:** 2012-02-01

**Authors:** Anna Lisa Crowley, Han W Kim, Michele Parker, Deneen Spatz, Brenda Hayes, Lubna Bhatti, Christoph J Jensen, Jessica Ngo, John A Papalas, Patrick Pun, John P Middleton, Robert M Judd, Raymond J Kim

**Affiliations:** 1 Duke University Medical Center, Durham, NC, USA

## Summary

The incidence of nephrogenic systemic fibrosis in subjects receiving gadoversetamide for cardiovascular magnetic resonance is low (0.026% overall and 1.274% in those subjects with CKD Stage 5 on hemodialysis).

## Background

Since 2006, the US Food and Drug Administration (FDA) has recommended restricting gadolinium based contrast agents (GBCAs) for magnetic resonance in patients with renal impairment due to an association of GBCA use with nephrogenic systemic fibrosis (NSF). The multiple FDA warnings have listed different glomerular filtration rate (GFR) cut-off values for restricting GBCA use. Consequently, hospital policies vary in renal impairment screening criteria and restrictions for both agent-specific and GFR cut-off values for GBCA use. Determining the incidence of developing NSF after exposure to specific GBCAs, and when stratified by chronic kidney disease (CKD) stage, may clarify which agents and patients are at highest risk for developing NSF. Currently, the incidence of developing NSF after exposure to gadoversetamide (Covidien, Mansfield MA) is unknown. The objective of this study was to determine the incidence and patients at highest risk of developing NSF in a large cohort of patients with suspected cardiovascular disease receiving gadoversetamide.

## Methods

Enrolled adult patients (n=7653) received gadoversetamide for a cardiovascular magnetic resonance (CMR) imaging study at Duke University Medical Center between July 1, 2002 and July 31, 2008. All database creatinine and gadoversetamide doses were verified by examining the original documentation at the time of the CMR. Multiple, redundant search strategies were performed in parallel to identify subjects with NSF including interrogation of Duke pathology, nephrology, and other available clinical databases. All search strategies were cross-matched against one another to verify that the same patients were identified using each strategy. For patients with CKD Stages 4 (n=263) and 5 (n=174), all available medical records were individually examined with particular attention paid to nephrology, dermatology, rheumatology, and pathology encounters. Incidence of NSF and 95% confidence intervals were determined overall and stratified by CKD stage.

## Results

**Figure 1:**
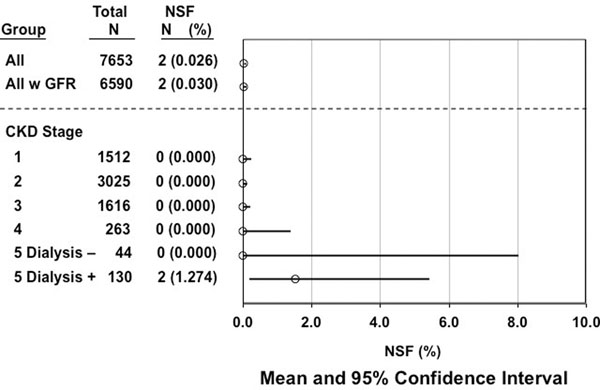
Incidence of NSF in subjects receiving gadoversetamide for cardiovascular magnetic resonance.

Two patients developed NSF. See figure 1.

## Conclusions

The incidence of developing NSF after exposure to gadoversetamide is low (overall: 0.026%; CKD Stage 5 on hemodialysis: 1.274%) in the largest cohort to date examining gadoversetamide. Both NSF patients had CKD Stage 5 on hemodialysis. Zero patients with Stage 5 CKD not on hemodialysis developed NSF. The wide 95% confidence interval for the latter group is likely due to the small number of patients (n=44). Further review is needed to determine if gadoversetamide should be less restricted in patients with moderate renal impairment in order to offer diagnostic capabilities to those patients who may suffer detrimental effects due to delayed or missed diagnoses.

## Funding

Covidien.

